# Neurocognitive Correlates of Psychosocial Functioning in Euthymic Bipolar Disorder: A Cross-Sectional Study

**DOI:** 10.1192/j.eurpsy.2026.11220

**Published:** 2026-07-31

**Authors:** B. Aifa, M. A. Yaich, S. Ben Othmen, H. Memi, A. Aissa

**Affiliations:** Mental Health, Nabeul Regional Hospital, Nabeul, Tunisia

## Abstract

**Introduction:**

Psychosocial functioning remains impaired for many individuals with bipolar disorder (BD) even during clinical remission. We aimed to describe psychosocial functioning in euthymic BD and isolate the contribution of neurocognitive deficits to functional outcomes.

**Objectives:**

Describe psychosocial functioning (FAST total and domains) in euthymic bipolar disorder. Assess key cognitive domains: verbal learning, semantic memory, visuospatial working memory, sustained attention, cognitive flexibility.-Examine associations between cognitive performance and FAST outcomes (total and domain scores). Identify independent cognitive predictors of functional outcomes via regression (variance explained, R^2^). Inform targets for cognitive assessment and remediation to improve everyday functioning.

**Methods:**

In a descriptive, analytic cross-sectional study, 60 euthymic adults with BD I, BD II, or other specified BD completed the Functioning Assessment Short Test (FAST). A brief neuropsychological battery assessed verbal learning, semantic memory, visuospatial working memory, sustained attention, and cognitive flexibility. Associations between neurocognition and FAST total and domain scores (autonomy, occupational, cognitive, interpersonal) were examined; variance explained by cognitive predictors was estimated with R^2^.

**Results:**

Overall psychosocial functioning was impaired in 75% of participants, with the cognitive (82%) and occupational (78%) domains most affected. Greater impairment in autonomy was observed among patients with poorer verbal learning (p=0.038), semantic memory (p=0.021), visuospatial working memory (p=0.007), sustained attention (p=0.045), and cognitive flexibility (p=0.039). In regression models, sustained attention (R^2^=0.105) and visuospatial working memory (R^2^=0.148) independently contributed to occupational functioning.

**Conclusions:**

In euthymic BD, deficits in attention, working memory, learning, memory, and cognitive flexibility are common and meaningfully related to poorer autonomy and occupational functioning. Systematic cognitive assessment and targeted cognitive remediation may improve everyday functioning during inter-episode periods.

**Disclosure of Interest:**

None Declared

